# Local endocytosis of sucrose transporter 2 in duckweed reveals the role of sucrose transporter 2 in guard cells

**DOI:** 10.3389/fpls.2022.996618

**Published:** 2022-10-24

**Authors:** Penghui Liu, Yang Fang, Xiao Tan, Zhubin Hu, Yanling Jin, Zhuolin Yi, Kaize He, Cuicui Wei, Rui Chen, Hai Zhao

**Affiliations:** ^1^ CAS Key Laboratory of Environmental and Applied Microbiology, Environmental Microbiology Key Laboratory of Sichuan Province, Chengdu Institute of Biology, Chinese Academy of Sciences, Chengdu, China; ^2^ University of Chinese Academy of Sciences, Beijing, China

**Keywords:** duckweed, sucrose transporter protein, guard cells, local endocytosis, ROS

## Abstract

The local endocytosis of membrane proteins is critical for many physiological processes in plants, including the regulation of growth, development, nutrient absorption, and osmotic stress response. Much of our knowledge on the local endocytosis of plasma membrane (PM) protein only focuses on the polar growth of pollen tubes in plants and neuronal axon in animals. However, the role of local endocytosis of PM proteins in guard cells has not yet been researched. Here, we first cloned duckweed SUT2 (sucrose transporter 2) protein and then conducted subcellular and histological localization of the protein. Our results indicated that LpSUT2 (*Landoltia punctata* 0202 SUT2) is a PM protein highly expressed on guard cells. *In vitro* experiments on WT (wild type) lines treated with high sucrose concentration showed that the content of ROS (reactive oxygen species) in guard cells increased and stomatal conductance decreased. We observed the same results in the lines after overexpression of the *LpSUT2* gene with newfound local endocytosis of LpSUT2. The local endocytosis mainly showed that LpSUT2 was uniformly distributed on the PM of guard cells in the early stage of development, and was only distributed in the endomembrane of guard cells in the mature stage. Therefore, we found the phenomenon of guard cell LpSUT2 local endocytosis through the changes of duckweed stomata and concluded that LpSUT2 local endocytosis might be dependent on ROS accumulation in the development of duckweed guard cells. This paper might provide future references for the genetic improvement and water-use efficiency in other crops.

## Highlights

1. ROS content in guard cells of overexpressed lines was higher than that in WT duckweed lines.
2. The LpSUT2:eGFP fusion protein was polarity distributed in duckweed guard cells. It was only located on guard cells’ endomembrane in the mature fronds while evenly distributed on both the outer membrane and endomembrane of guard cells in the young fronds.
3. The ROS-dependent local endocytosis occurs on guard cells LpSUT2 protein during the development of overexpression lines, accompanied by the closing of stomata.
4. LpSUT2 protein is a promising candidate gene for drought improvement in crops.

## Introduction

Endocytosis, the major route of PM (plasma membrane) proteins to enter cells, is an evolutionarily conserved eukaryotic pathway that internalizes extracellular substances and PM components through vesicles (Johnson et al., 2021; Su et al., 2021). Endocytosis plays a central role in many developmental and physiological processes in plants by regulating the abundance and activity of PM proteins (Wang et al., 2021; Wang et al., 2022), especially during the polar growth of cells (Li et al., 2018) or under hyperosmotic stress (Martinière et al., 2019).

Osmotic stress exerts strong and rapid impacts on cell membrane dynamics (Martinière et al., 2019). Endocytosis of PM proteins is crucial to maintaining plant cells’ osmotic balance. There are two types of endocytosis: constitutive and local. Recent studies have shown that the aquaporin PIP2;1 of *Arabidopsis thaliana* root cells and guard cells regulate intracellular osmotic balance *via* constitutive endocytosis under hyperosmotic stress (Boursiac et al., 2008; Wudick et al., 2015; Martinière et al., 2019; Cui et al., 2021). Similarly, constitutive endocytosis renders the PIN (PIN-FORMED) auxin efflux transporters polar distribution in *Arabidopsis thaliana* root cells, an important process for regulating the directional flow of auxin (Dhonukshe et al., 2007; Kleine Vehn et al., 2011; Łangowski et al., 2016). In addition, the endocytosis of PM proteins is critical for nutrient absorption. For example, boron (B) is essential for plant growth but poisons the plant when presented in excess. When this happens, the activity of borate exporters BOR1 and boric acid channel NIP5;1 protein in *Arabidopsis thaliana* root cells would be suppressed through endocytosis to avoid the plants’ B poisoning (Takano et al., 2010). On the other hand, the local endocytosis of plant PM proteins regulates the establishment and maintenance of cell polarity. For instance, Hui Li et al. concluded that clathrin-mediated local endocytosis affects the activity of REN4 (located at lateral)/ROP1 (located at apex) and plays a critical role in the polar growth of *Arabidopsis thaliana* pollen tube (Li H. et al., 2018). Analogous local endocytosis also exists in fungal hyphae growth (Schultzhaus and Shaw, 2015), plant root hair cells elongation (Zhu et al., 2020; Kuběnová et al., 2022), and animal neuronal axon (Kanamori et al., 2015; Zhu et al., 2020). The guard cells share the same polar growth pattern to plant root hair and pollen tube (Carter et al., 2017; Lopez Anido et al., 2021; Ramalho et al., 2021), However, local endocytosis of PM proteins in guard cells has not yet been discovered. Transcriptomic and proteomic data showed that SUT protein was highly expressed in the PM of guard cells, the protein responsible for transporting mesophyll derived sucrose into guard cells and participating in stomatal movement (Daloso et al., 2016). Hence, we studied the local endocytosis of guard cells with the SUT membrane protein of duckweed.

Duckweed comprises 36 species from 5 genera (*Spirodela, Landoltia, Lemna, Wolffiella*, and *Wolffia*) (Bog et al., 2020), is a tiny aquatic plant. It can double its biomass in 1-4 days, and grows much faster than most other higher plants (Ziegler et al., 2015; Michael et al., 2020). In addition, duckweed can accumulate high starch (40-50%) under specific culture conditions (Sree et al., 2015; Acosta et al., 2021; Sree and Appenroth, 2022). Interestingly, there are few reports on the stomata of duckweed. In contrast to other plants, stomata remain open in duckweed even upon prolonged exposure to the phytohormone abscisic acid (McLaren and Harry, 1976; Acosta et al., 2021), light, temperature, and carbon dioxide. Our work found that LpSUT2:eGFP protein was evenly distributed in guard cells of young fronds but only located at guard cells endomembrane of the mature fronds, also known as polarity distribution in duckweed guard cells. This might be caused by the up-regulation of *LpSUT2* expression that leads to higher sucrose stress in guard cells, generating the signal molecule ROS and stimulating LpSUT2 local endocytosis of guard cells. Meanwhile, the change in the overexpressed lines’ stomatal behavior might support the occurrence of local endocytosis of LpSUT2. This paper concluded that local endocytosis occurs on LpSUT2 protein during the development of overexpression lines guard cells, accompanied by the closing of stomata. Therefore, LpSUT2 might be used as a target gene to improve water-use efficiency for other crops.

## Materials and methods

### Plant materials and growth conditions

*Landoltia punctata* 0202 strain and *Lemna minor* (M0157) callus were stored in the duckweed resource bank at the Chengdu Institute of Biology, Chinese Academy of Science (Tian et al., 2021). Duckweed was inoculated in a sterilized and modified Hoagland medium with 1.5% (m/v) sucrose (pH 5.00 ± 0.05) under a 16 h light/8 h dark cycle with a photon flux density of 100-120 μmol^-2^s^-1^. The sample was cultured for two weeks at a temperature cycle of 25°C/15°C (day/night), then used as succeeding experimental materials.

### Sequence and phylogenetic analysis

LpSUT2 protein transmembrane structure was analyzed using Phobius (https://phobius.sbc.su.se/) and Gnuplot software (command-driven interactive function plotting program). Multiple sequence alignments were generated using MEGA 6.0 software. The neighbor-joining method was used for constructing the phylogenetic tree based on bootstrap analysis for 500 replications. Subsequently, the graphic was optimized through AI (adobe illustrator) software.

### Yeast complementation assay

For heterogeneous expression in *Saccharomyces cerevisiae* (SEY6210), we amplified the CDS sequence that translates to LpSUT2 protein. The recombinant primer pairs were 5’- *TCGACTAGTGGATCC* CCCGGGATGGTCAGCGTCCAAGATCTGGAT-3’ (Forward) and 5’- *GGTACCGGGCCCCCC* CTCGAGTCAGCCAAACCCATGGAACCCTGAC-3’ (Reverse). The underlined sequences indicated the introduced restriction enzyme sites (*Sma*I and *Xho*I), and italic sequences represented the overlapping area. Additionally, the pDR196 vector was digested with *Sma*I and *Xho*I. The previously amplified products and the linearized vector were connected by recombinase and then sequenced. Furthermore, we modified the N-terminal amino acid sequence of LpSUT2 protein with reference to the previous methods (Schulze et al., 2000): the first 51 amino acids (the first intracellular domain) of LpSUT2 were exchanged with the respective N-terminal 28 amino acids sequence of StSUT1 (the first intracellular domain), then the chimeric construct was named LpSUT2N. The pDR196:LpSUT2 vector, pDR196:StSUT1 (positive control), and pDR196:LpSUT2N were then transformed into the yeast strain SEY6210 for sucrose uptake analysis.

The process of vector transforming yeast is as follows. Inoculate cells from a single colony into 20 mL YPDA and incubate at 30°C until OD_600 =_ 1-2. Transfer enough of this culture to a 300 mL YPDA medium in a 1 L flask to produce an initial OD_600_ of 0.2. Incubate with shaking (250 rpm/min) for 3 h. Centrifuge cells at 1000 g for 5 min at RT (room temperature). Discard the supernatant and resuspend cells pellet in 50 mL H_2_O. Centrifuge cells again (same conditions as above), discard the supernatant, and resuspend the pellet in 1.5 mL sterile LiAc solution. Set up the required number of Eppendorf tubes and add 100 μL of cells. Add 0.1 µg of each type of plasmid, together with 100 μg of salmon sperm DNA. Add 0.6 mL sterile PEG/LiAc solution to each tube and vortex. Incubate at 30°C for 30 min with shaking (250 rpm/min). Add 70 μL of 100% DMSO (dimethyl sulfoxide) and mix gently. Heat shock for 15 min at 42°C. Chill cells on ice and pellet in a centrifuge for 5 s at 14000 rpm/min. Remove the supernatant and resuspend cells in 0.5 mL of TE buffer. Spread 100 μL of this mixture onto each standard size plate containing the appropriate selection of medium. Incubate plates for 3-4 days until colonies appear.

### Subcellular localization assays

A transient expression assay of LpSUT2 using protoplasts from duckweed was performed in this work. To obtain recombinant plasmids, we cloned the full-length cDNA of the *LpSUT2* gene (1710 bp) by recombinant primer 5’-GTCCTGCAGTGCCATAAGCTTATGGTCAGCGTCCAAGATCTGGATA-3’ and 5’-CACCATAGATCTGCCAAGCTTGCCAAACCCATGGAACCCTGACATG-3’ and amplified with used high PCR (polymerase chain reaction) fidelity KOD Plus enzyme (TOYOBO, Japan). The thermal cycle program is as follows: 2 min at 94°C, 35 cycles of (10 s at 98°C, 30 s at 60°C, 120 s at 68°C), and 5 min at 68°C. PCR products were inserted into the binary vector pCambia2301 containing the cauliflower mosaic virus 35S promoter and the eGFP (enhancer green fluorescent protein) on restriction enzyme sites *Hind*III. According to a previously established method, the recombinant vector was introduced into duckweed callus using *Agrobacterium tumefaciens* strain GV3101-mediated transformation (Yamamoto et al., 2001). The callus of duckweed was co-cultured with *Agrobacterium tumefaciens* strain GV3101 that carried either the target vector (pCambia2301:35S:LpSUT2:eGFP) or control vector (pCambia2301:35S:eGFP). They were cultured for two days in an MS medium containing 30 g/L sucrose, 1 μM 2,4-Dichlorophenoxyacetic acid, 2 μM 6-Benzylaminopurine, and 100 μM Acetosyringone. Next, following a previous research method (Yoo et al., 2007), cellulase R-10 enzyme, macerozyme R-10, and pectolyase Y-23 enzyme solution with the co-cultured callus generated duckweed protoplasts. Then the fluorescence signal of eGFP was observed with a laser scanning confocal microscope (excitation filter, 488 nm; emission filter bandpass, 505-530 nm, Leica TCS SP8).

### Promoter activity assays

The 2000 bp promoter sequence upstream of the *LpSUT2* was extracted from the genome of *L. punctata* by TBtools software. To analyze the activity of the *LpSUT2* promoter, we transiently expressed the proLpSUT2:eGFP vector in *Nicotiana tabacum*, and the eGFP fluorescence intensity was observed using a laser scanning confocal microscope (excitation filter, 488 nm; emission filter bandpass, 505-530 nm).

### Histological localization assays

The pCambia2301:proLpSUT2:eGFP vector was constructed by cloning PCR-amplified fragments containing a 2000 bp sequence of the *LpSUT2* promoter region. The following recombinant primers were used to amplify DNA fragments for the *LpSUT2* promoter: the proLpSUT2-F (5’-GATTCATTAATGCAGCTGAGCTCCCTCTCCTTCTTCTCCT-3’) and proLpSUT2-R (5’-GCCAAGCTTATGGCACTGCAGCATCTCCAATTCAGCCTCC-3’). These PCR fragments were ligated into the linearization (digested with *Pvu*II and *Pst*I) binary vector pCambia2301:eGFP (modified from pCambia2301:GUS) by ClonExpress^®^ MultiS One Step Cloning Kit (Vazyme, Nanjing, China) and then sequenced, resulting in a vector with eGFP fused on the C-terminus of the *LpSUT2* promoter. Duckweed (*Lemna minor*) callus induction and transformation followed the protocol described (Yamamoto et al., 2001; Cox et al., 2006). Then eGFP fluorescence signal was observed with a laser scanning confocal microscope (excitation filter, 488 nm; emission filter bandpass, 505-530 nm).

### Overexpression of *LpSUT2*

The pCambia2301:LpSUT2:eGFP vector was constructed and then ligated into the linearization (digested with *Hind*III) binary vector pCambia2301:eGFP (modified from pCambia2301:GUS) by ClonExpress^®^ MultiS One Step Cloning Kit (Vazyme, Nanjing, China) and then sequenced. Subsequently, gene transformation, selection, and frond regeneration followed the protocol described (Yamamoto et al., 2001; Cox et al., 2006).

### Microscopic observation of stomata

WT (wild type) and overexpressed duckweed were cultivated in Hoagland medium for three days, then sampled. We detected the stomata in more than 100 duckweed lines (overexpressed lines and WT lines) with a laser scanning confocal microscope (Equipment, Leica TCS SP8; Software, LAS AF). Then we measured the stomatal aperture length of duckweed (overexpressed lines and WT lines) by ImageJ software.

### Relative fluorescence intensity

Guard cells and epidermal cells were further examined with a laser scanning confocal microscope (Equipment, Leica TCS SP8; Excitation filter, 450-490 nm; emission filter bandpass, 520-560 nm; Software, LAS AF; Values of intensity, 30%; the maximum master gain, 800; Digital gain, 1). The relative fluorescence density of at least 40 guard cells and epidermal cells was quantified with Leica TCS SP8 software. Operation was performed as follows: Quantify-Tools-Intensity-Draw polygon-Export data. Subsequently, we drew violin plot figures with GraphPad prism 8.0.2 software.

### ROS measurement

To detect ROS (reactive oxygen species) in guard cells, 50 μM fluorescent probes H_2_DCF-DA (2’7’-dichlorodihydrofluorescein diacetate) (Coolaber, Beijing, China) was used, with only slight modifications from previous studies (Khokon et al., 2011; Li et al., 2016). Duckweed frond was incubated under darkness at room temperature in Tris-KCl buffer (10 mM Tris and 50 mM KCl, pH7.2) with 50 μM H_2_DCF-DA for 30 min and then washed three times with fresh Tris-KCl buffer to remove the residual probes. Subsequently, the fluorescence signal of guard cells was observed with a laser scanning confocal microscope (Excitation filter, 450-490 nm; emission filter bandpass, 520-560 nm) (Leica TCS SP8).

### Isolation of RNA and qRT−PCR analysis

Total RNAs were extracted using Eastep Super Total RNA Extraction Kit (Promega, Shanghai, China) according to the manufacturer’s instructions. Next, we examined their concentration and quality with NanoDrop 2000 (Thermo, USA). All RNA samples were treated with RNase-free DNase to eliminate DNA contamination. The cDNA was synthesized from 1 μg total RNAs following the manufacturer’s protocol of GoScript™ Reverse Transcription System (Promega, Shanghai, China). These samples were harvested and immediately frozen in liquid nitrogen, and subsequently stored in a -80°C freezer until use. The expression of the primer pairs of LpSUT2 (5’-CCGAGGCTTGTTTGGGCTTTGA-3’ and 5’-ATGTGCTGGACGCTCTCTGAGT-3’) as well as that of *actin* (5’-TGATGGTTGGAATGGGACAG-3’ and 5’-TTGGTCACAACGCCATGCT-3’) of *L. punctata* as the reference gene for normalization, were used to analyze the expression level. The qRT-PCR (Real-time fluorescence quantitative PCR) was operated with the SsoAdvanced™ Universal SYBR Green Supermix (Bio-Rad, USA) using CFX Maestro Real-Time PCR (Bio-Rad, USA). The statistical analysis was performed on expression level using the t test in GraphPad Prism 8.0.2 and the results were shown as mean values ± SD of three independent experiments (P value<0.001). The PCR reaction system, procedures, and data analysis followed SsoAdvanced™ Universal SYBR Green Supermix protocol.

### Sucrose content measurement

A detailed description of the sucrose extraction method can be found in Zhu et al. (Zhu et al., 2018). The sucrose content was determined using an evaporative light-scattering detector (All-Tech ELSD 2000, All-tech, Crop, USA) of HPLC (high-performance liquid chromatography) system (Thermo 2795, Thermo Corp, USA) and statistically analyzed as above.

## Results

### Identification of the *LpSUT2* gene and bioinformatics analysis

**Figure 1:**
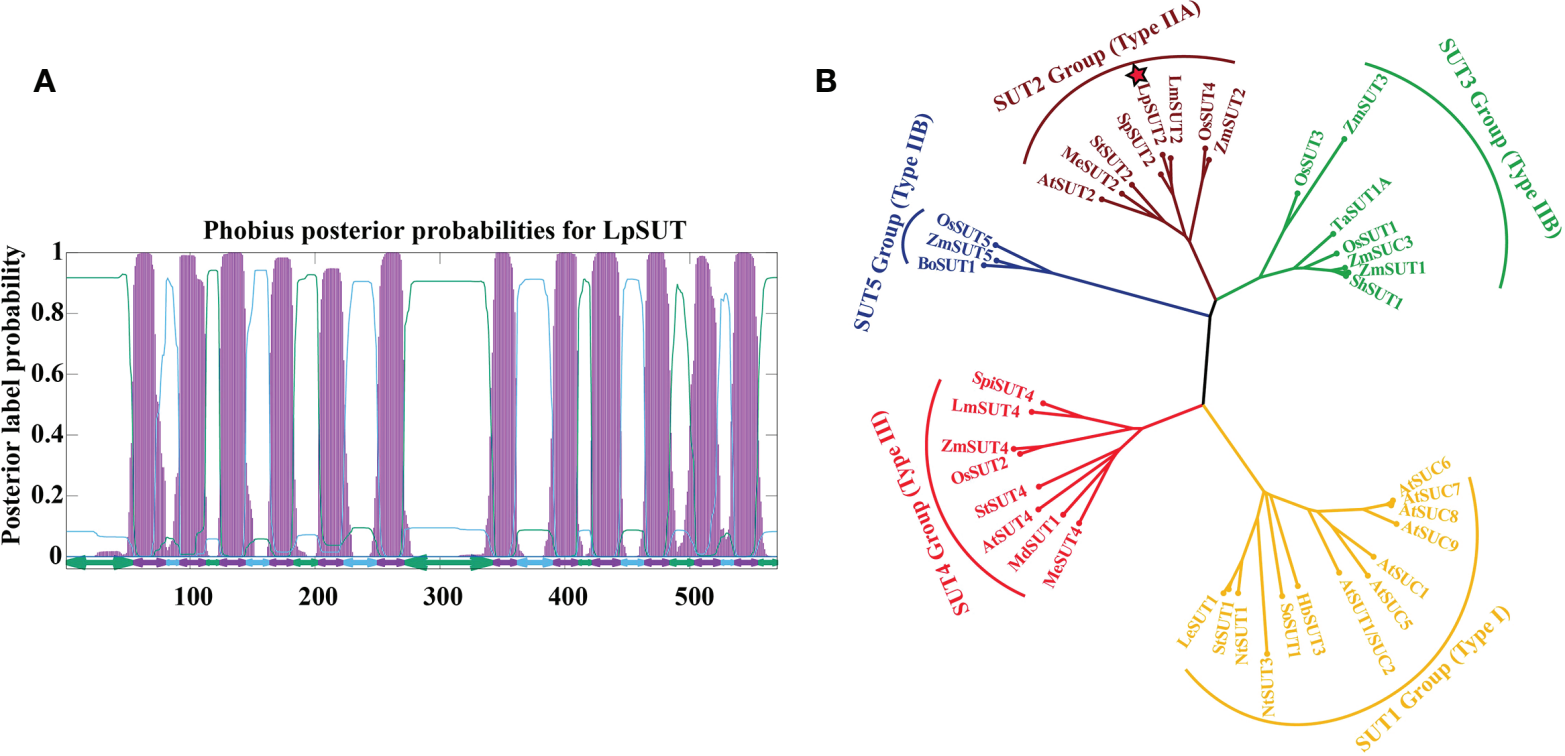
Sequence analyses of the LpSUT2. **(A)** 12 transmembrane domains were predicted in LpSUT2. The figure was produced using the Phobius server (https://phobius.sbc.su.se/) and Gnuplot software. **(B)** Phylogenetic tree was constructed using the analysis of the LpSUT2 and SUTs proteins from other plant species. Sequence names and accession numbers are provided in **Table S1**. The bootstrap consensus tree from 500 replications was created based on the NJ (neighbor-joining) method, using MEGA 6.0. LpSUT2 sequence falls into IIA clades (SUT2 subfamily). LpSUT2, Sucrose transporter 2 of *L. punctata*.

In this research, the cDNA of *LpSUT2* was cloned from *L. punctata* (*Landoltia punctata* 0202) by PCR with specific primer pairs LpSUT2-F and LpSUT2-R. The CDS (complete coding sequence) of *LpSUT2* is 1710 bp long (approximately a 60.91 kD protein). The structural prediction of membrane domains showed that the protein had 12 transmembrane domains (**Figure 1A**). To evaluate the evolutionary relationships between LpSUT2 protein and the identified SUTs from other species, we constructed a phylogenetic tree with 39 SUT sequences from 15 plant species (**Figure 1B**). It concluded that LpSUT2 (marked by red stars) belonged to the Type IIA clade (SUT2 subfamily) (**Figure 1B**). In addition, LpSUT2 protein, unlike other members of the SUT subfamily, has a long central cytoplasmic loop composed of 64 amino acids and contains the extended N-terminus (Barker et al., 2000). The similarity found between LpSUT2 and members of the SUT2 subfamily (**Figure S1**) led to the naming of the *L. punctata* sucrose transporters 2 gene as *LpSUT2*. These results indicated that *LpSUT2* shared a similar structure to the *SUT2* group member, which is low affinity sucrose transporters (Cai et al., 2020) and is a putative sucrose sensor in sieve elements (Barker et al., 2000). For example, the AtSUT2 protein of *Arabidopsis thaliana* is mainly responsible for the loading of sucrose in phloem and the recovery of leaked sucrose in long distance transportation (Srivastava et al., 2008; Gould et al., 2012). Therefore, we speculated that the LpSUT2 have similar functions.

### Expression of LpSUT2 in yeast

**Figure 2:**
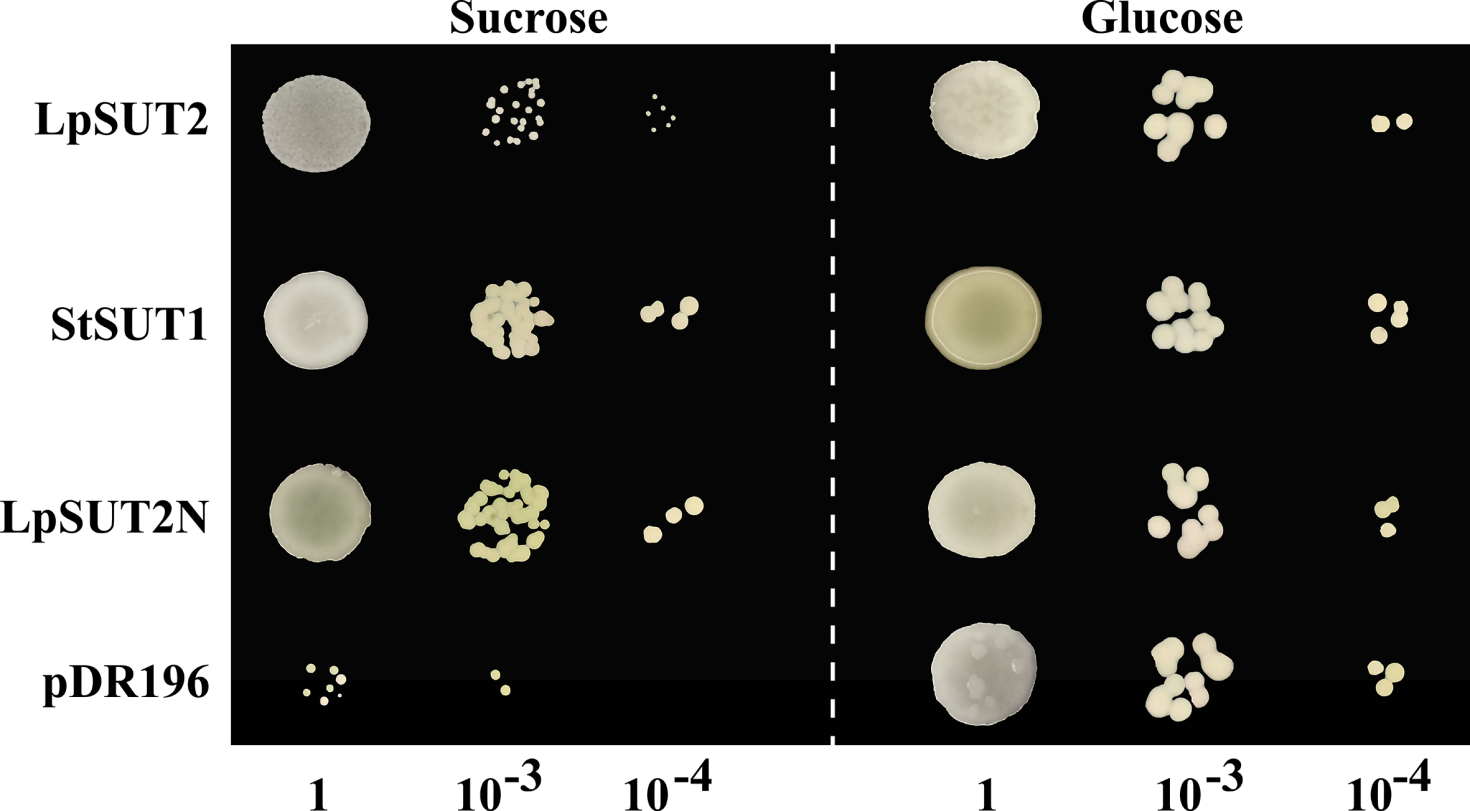
Functional analysis of LpSUT2 protein by yeast complementation assay. Sucrose uptake-deficient mutant yeast strains SEY6210 transformed with LpSUT2, LpSUT2N, StSUT1 (positive control), and pDR196 empty vector (negative control) were grown on a medium of 2% sucrose and 2% glucose (positive control) as sole carbon source. The plates were incubated at 30°C for 2-3 days, and the colonies were observed at different dilution times. LpSUT2, Sucrose transporter 2 of *L. punctata*; StSUT1, Sucrose transporter 1 of *Solanum tuberosum*.

To verify whether the LpSUT2 protein is a functional sucrose transporter, we inserted the full-length CDS sequences of *LpSUT2* and *StSUT1* (*Solanum tuberosum*) into the yeast expression vector pDR196 which was subsequently transformed into sucrose uptake deficient yeast mutant strains SEY6210 (**Figure 2**). Clear complementary colonies were observed as the yeast strains expressed LpSUT2 and StSUT1 protein (positive control) when grown on a selective medium with exclusive carbon sources of 2% sucrose or 2% glucose (positive control). Compared with the positive control, the yeast expressing LpSUT2 protein grows slower and has a smaller morphology. In contrast, expression of the empty vector pDR196 (negative control) hardly restores yeast growth (**Figure 2**). Further, after modifying the N-terminal amino acid sequence of LpSUT2, we observed significant complementation in the yeast strains expressing LpSUT2N protein. This means that the transport capacity of LpSUT2 is weak. This illustrates that LpSUT2 protein is a low-affinity sucrose transporter like AtSUT2 (Schulze et al., 2000; Kühn, 2003). Our results showed that the N-terminal amino acid sequence of LpSUT2 protein may be important for determining substrate affinity, which was consistent with the result of modifying the N-terminal amino acid sequence of AtSUT2 protein (the N-terminus of AtSUT2 showed significantly lower affinity for sucrose compared to the N-terminus of StSUT1) (Schulze et al., 2000). In addition, we predicted the binding site of LpSUT2 protein and sucrose by CB-Dock database (http://clab.labshare.cn/cb-dock/php/manual.php) (Liu et al., 2020) (**Figure S2**). The results indicated that several key amino acids (V2, L7, N10, S11, E12, D14, S18, L24, P25, I26, S27, L41, V44) in the N-terminal domain might affect the function of LpSUT2 protein, which needs further research.

### Subcellular and histological localization of LpSUT2

**Figure 3:**
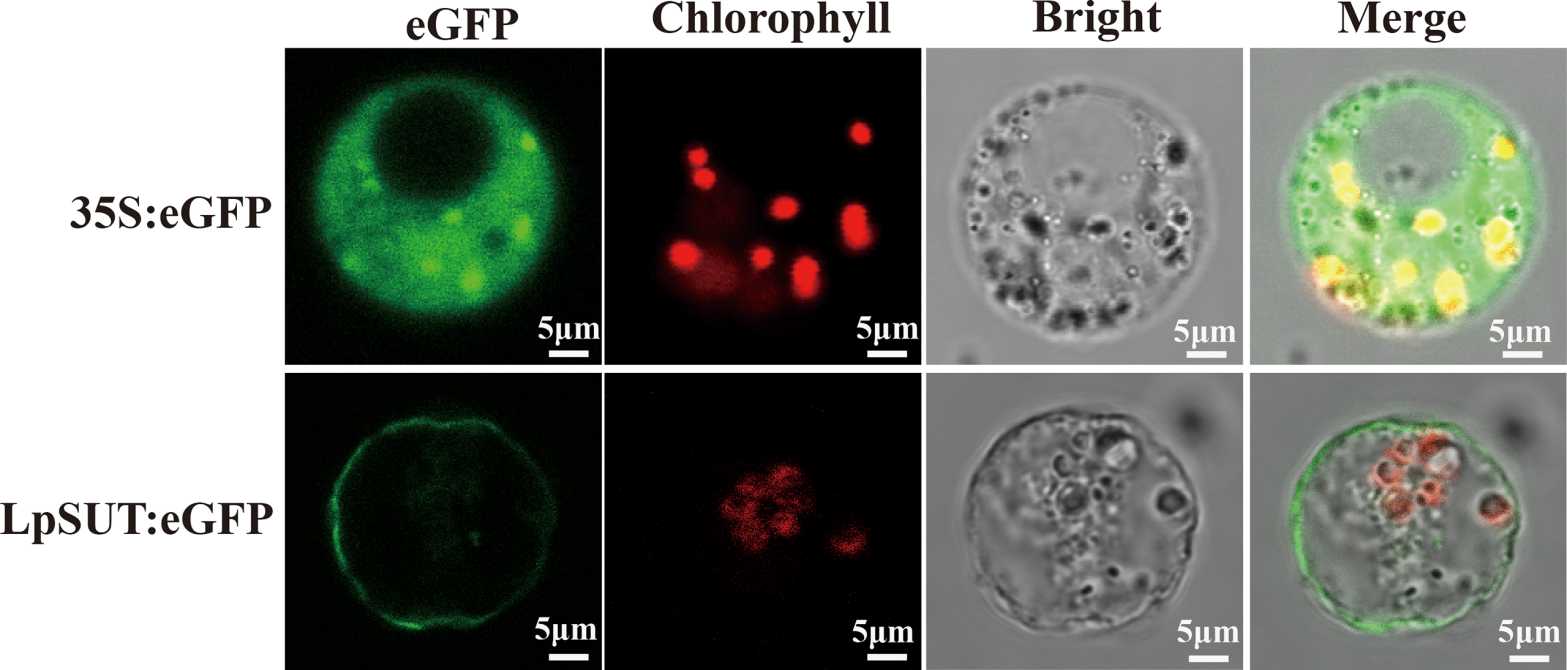
Subcellular localization of the LpSUT2. Transient expressions of the binary vector pCambia2301:eGFP and pCambia2301:LpSUT2:eGFP in the protoplast were observed under a laser scanning confocal microscope (excitation filter, 488 nm; emission filter bandpass, 505-530 nm). LpSUT2:eGFP fusion proteins were found located on PM. The eGFP protein is indicated in green, and chlorophyll autofluorescence is indicated in red. The eGFP, chlorophyll auto-fluorescence, bright field, and merged images were presented. Scale bar = 5 μm. LpSUT2, Sucrose transporter 2 of *L. punctata*; eGFP, Enhanced green fluorescent protein.

Several studies agree that plant IIA clade SUTs are localized to the PM (Reinders et al., 2012). We constructed a C-terminal translational fusion of eGFP (enhancer green fluorescent protein) to LpSUT2 under the Cauliflower Mosaic Virus 35S promoter (pCambia2301:35S:LpSUT2:eGFP). Positive control (pCambia2301:35S:eGFP) was ubiquitously expressed in the cytoplasm. Confocal images of protoplasts demonstrated that the LpSUT2:eGFP fusion protein was distributed along with the PM (**Figure 3**). Furthermore, a dileucine-like motif (LXXXLL) in the N-terminal cytoplasmic domain of the *Arabidopsis* monosaccharide transporter ESL1 was shown to be necessary for the localization of the transporter to the vacuole membrane (Yamada et al., 2010; Reinders et al., 2012). The conservative LXXLL motif is found in the cytoplasmic N-terminal of type III SUTs (except for AtSUC4, which has the sequence KRVLL, also localized to the vacuole membrane) (Wang et al., 2020) but is not found in type I and type II SUTs (**Figure S3**). Sequence analysis of the LpSUT2 revealed no conserved LXXLL motif in the cytoplasmic N-terminus. Therefore, based on the results of the conservative LXXLL motif and subcellular localization, we suspected that LpSUT2 is a PM-localized protein.

**Figure 4:**
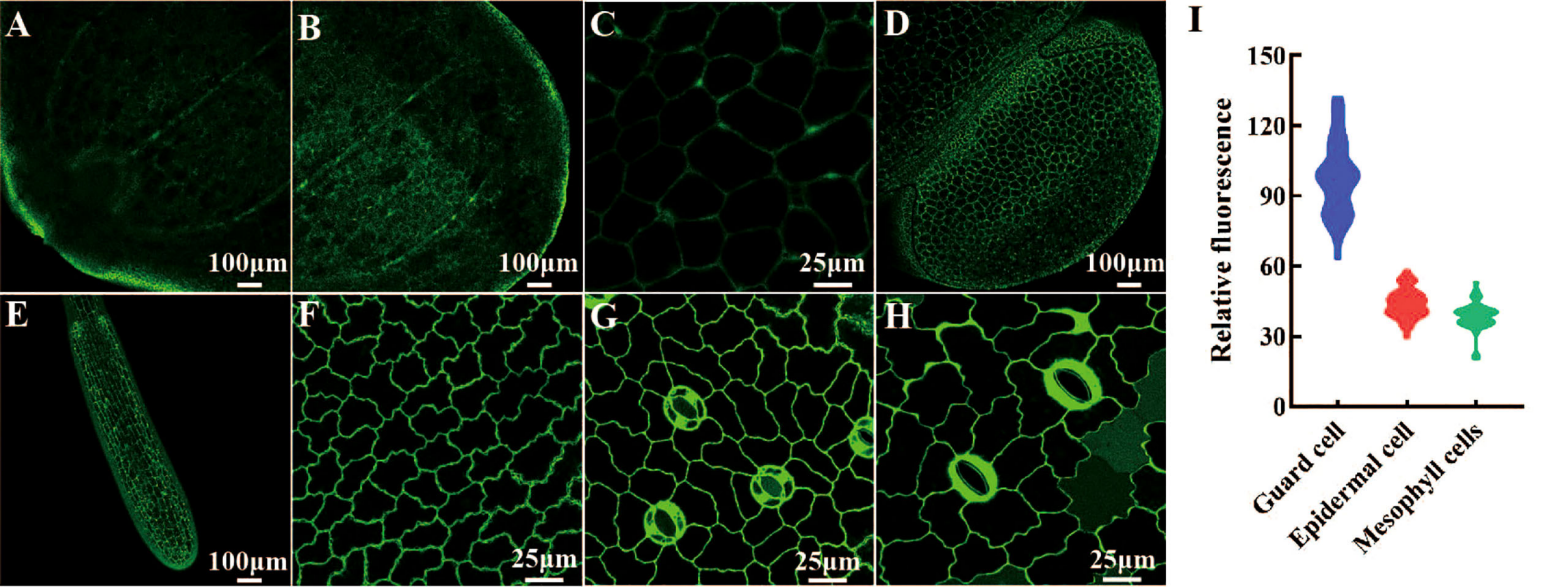
Histological localization of LpSUT2. The pCambia2301:proLpSUT2:eGFP transgenic duckweed expressing eGFP is generated and eGFP fluorescence protein is detected in all tissue cells of transgenic duckweed fronds, including veins **(A-B)**, parenchyma cells **(C)**, mature fronds and young fronds **(D)**, root cells **(E)**, epidermal cells **(F)**, and guard cells **(G-H)**. **(I)** The relative fluorescence intensity of eGFP in different cells (at least 40 cells respectively) with a laser scanning confocal microscope. Scale bars: 100 μm in **(A, B, D, E)**; 25 µm in **(C, F, G, H)**. LpSUT2, Sucrose transporter 2 of *L. punctata*; eGFP, Enhanced green fluorescent protein.

We determined the histological expression profiles of LpSUT2 protein in duckweed fronds. We first generated pCambia2301:proLpSUT2:eGFP (the activity of *LpSUT2* gene promoter is almost equal to that of 35S promoter, **Figure S4**) transgenic duckweed expressing eGFP reporter. Histological localization indicated that LpSUT2 protein is expressed in all tissues of both young and mature duckweed fronds (**Figure 4A-H**), including epidermal cells, mesophyll cells, veins, root cells and guard cells, a pattern similar to AtSTP4 (Buttner, 2010), DcSUT2 (Shakya and Sturm, 1998), and AtSUC3 (Meyer et al., 2004). In addition, we measured the relative fluorescence intensity of eGFP with a laser scanning confocal microscope (**Figure 4I**). Interestingly, we noted that LpSUT2 is strongly expressed in guard cells, in accordance with previous results of transcriptomic and proteomic data (Bauer et al., 2013; Daloso et al., 2016).

### Overexpression of the LpSUT2 gene triggers stomatal behavior changes

**Figure 5:**
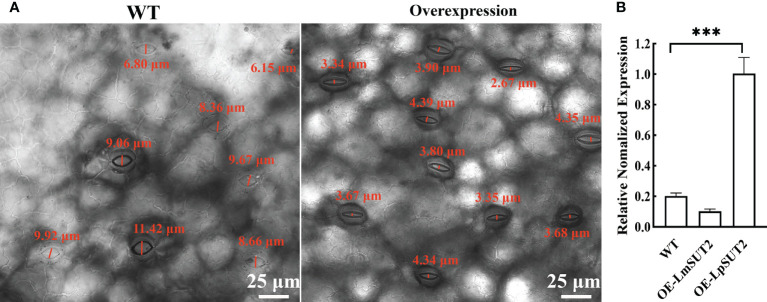
Effects of LpSUT2 on the stomata and analysis of LpSUT2 expression. The stomatal aperture of overexpressed lines decreased compared with that of WT **(A)**. Quantification of LmSUT2 and LpSUT2 mRNA accumulation is done by qRT-PCR analysis under a 16 h light/8 h dark cycle with a photon flux density of 100-120 μmol^-2^s^-1^ at a temperature cycle of 25°C/15°C (day/night) and is cultured for one week **(B)**. **Figure 5B** showed as mean values ± SD of three independent experiments (***P value<0.001). Scale bars: 25 μm in **(A)**. WT, Wild type; OE, Overexpression; LmSUT2, Sucrose transporter 2 of *Lemna minor*; LpSUT2, Sucrose transporter 2 of *L. punctata*.

**Figure 6:**
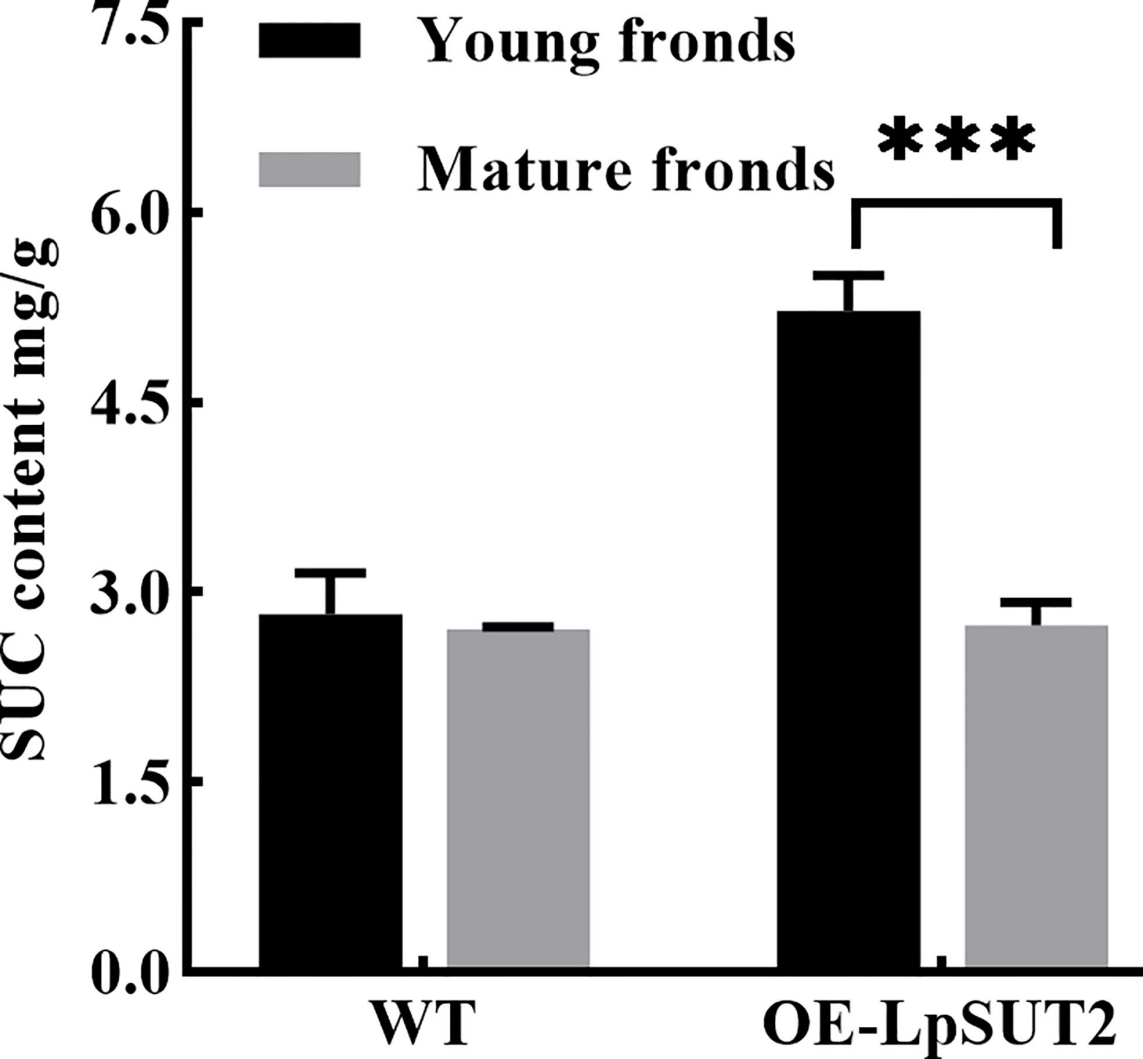
Variation of sucrose content during duckweed growth. The changes of sucrose content in WT and *LpSUT2* overexpression lines during its growth were determined using an evaporative light-scattering detector (All-Tech ELSD 2000, All-tech, Crop, USA) of HPLC (high-performance liquid chromatography) system (Thermo 2795, Thermo Corp, USA). The content of sucrose in the overexpressed *LpSUT2* lines significantly decreased from initial values of 5.02 to 2.61 mg/g during the development from young to mature fronds. The bars indicate the mean value ± SD of three independent experiments (***P < 0.001). WT, Wild type; LpSUT2, Sucrose transporter 2 of *L. punctata*; OE, overexpression.

Histological localization results revealed that LpSUT2 protein was enriched on the guard cells membrane. Therefore, we overexpressed the *LpSUT2* gene in *Lemna minor* to determine its function in duckweed guard cells. We detected the expression levels of *LmSUT2* genes in WT lines and expression levels of heterologous *LpSUT2* gene and endogenous *LmSUT2* gene in overexpressed lines respectively. The results of qRT-PCR showed that the expression of *LpSUT2* in overexpression lines was 4.89 folds higher than that in WT lines (**Figure 5B**), as well as the expression level of total SUT2 (heterologous *LpSUT2* and endogenous *LmSUT2*) in overexpressed lines was 5.39 folds that of LmSUT2 in WT lines. Interestingly, the stomata of WT lines are open under many treatment conditions (ABA, light-dark cycle, carbon dioxide, etc.). However, the stomata of more than 100 duckweed lines (overexpressed lines and WT lines) were observed in this study. Our results indicated that stomatal conductance of overexpression lines was reduced (< 5 μm) compared with WT lines (> 5 μm), and the number of stomatal aperture with less than 5 μm accounts for about 60% of the total number of stomatal in each overexpressed line (**Figure 5A**). In addition, we detected the changes in sucrose content levels in WT and in overexpressed lines during their growth. Due to the particularity of duckweed’s morphology and structure, it is difficult to separate guard cells with current technology. So, we evaluated the change of sucrose content in the whole duckweed. **Figure 6** showed a slight change in sucrose level in WT lines. However, the sucrose level in the lines of overexpressed LpSUT2 gene highly significant (P value<0.001) decreased from initial values of 5.02 to 2.61 mg/g during the development from young to mature fronds (**Figure 6**). These observations suggested that LpSUT2 might affect the stomatal behavior by leveling the sucrose content in guard cells.

### LpSUT2 local endocytosis depends on ROS

**Figure 7:**
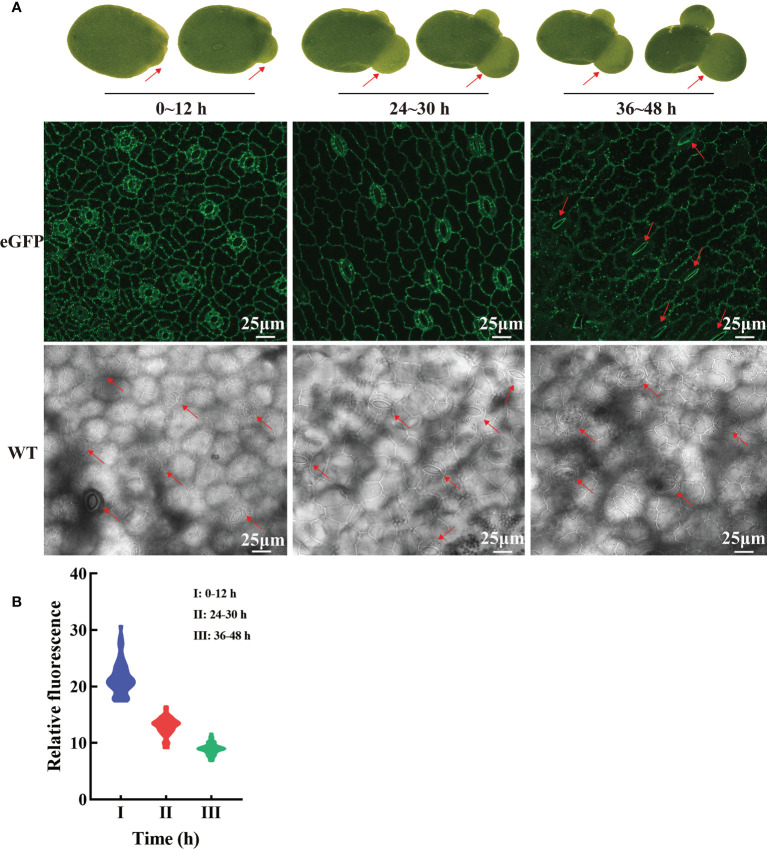
Dynamic changes of LpSUT2 subcellular localization in duckweed growth and development. In the initial stages of overexpressed fronds development, the LpSUT2:eGFP fusion protein is evenly distributed in the PM of the guard cells (0-12 h). It gradually formed polar distribution, with the LpSUT2:eGFP expressed only on the endomembrane at the mature stage (36-48 h) **(A)**. The relative fluorescence intensity of at least 40 guard cells was quantified with a laser scanning confocal microscope **(B)**. The red arrow points to the guard cell. Scale bars: 25 μm. PM, plasmalemma; LpSUT2, Sucrose transporter 2 of *L. punctata*; eGFP, Enhanced green fluorescent protein; WT, Wild type.

Hyperosmotic stresses (sugar stress, salt stress, drought stress et al.) induce ROS (reactive oxygen species) production and then impact the dynamic changes of PM protein. Our study investigated the subcellular localization changes of LpSUT2 protein during the development of guard cells. We found that LpSUT2:eGFP protein was asymmetrically distributed in duckweed guard cells: it was only located on guard cells’ endomembrane in the mature fronds while evenly distributed on both outer membrane and endomembrane of guard cells. The difference between mature and young fronds is the result of the local endocytosis of the LpSUT2 protein (**Figure 7A**). In addition, to examine the relative abundance of LpSUT2:eGFP protein in guard cells, we measured the relative fluorescence intensity of eGFP with a laser scanning confocal microscope (**Figure 7B**). The expression level of LpSUT2:eGFP significantly reduced with the development of guard cells (**Figure 7B**). At the same time, the stomatal conductance of overexpressed lines decreased during its development (**Figure 7A**). Generally, the direct cause of stomatal movement is the changes in osmotic pressures in guard cells such as sugar stress (Li Y. et al., 2018) directly causes stomatal movement.

**Figure 8:**
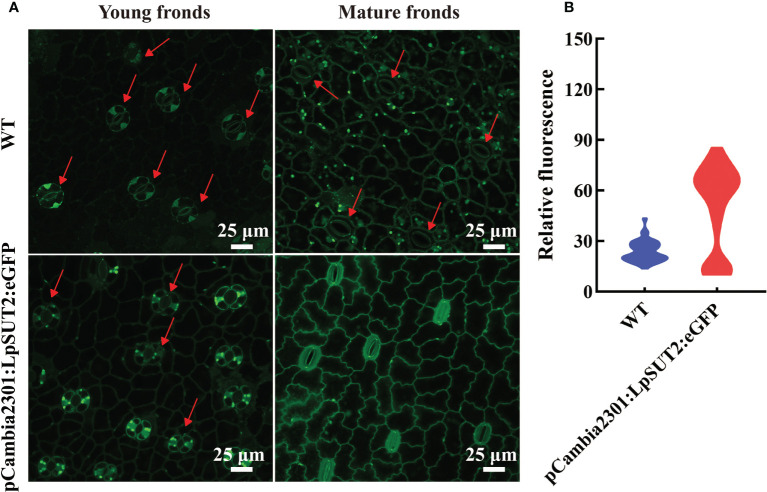
Detection of ROS accumulation in guard cells. The content of ROS in guard cells of young and mature fronds was detected in overexpressed lines and WT lines, respectively **(A)**. These results indicated that ROS content increased significantly and stomatal conductance decreased in guard cells of overexpression lines compared to that of WT lines **(A)**. The relative fluorescence intensity of at least 40 guard cells was quantified with a laser scanning confocal microscope **(B)**. The red arrow points to the guard cell. Scale bars: 25 μm in **(A)**. ROS, Reactive oxygen species; WT, Wild type.

We subsequently detected the content of ROS in guard cells because of its responsive nature to higher sucrose stress. ROS can be tested through loading fluorescent probe H_2_DCF-DA (Li et al., 2016). We detected the ROS in young and mature fronds in both WT and overexpressed lines to determine whether ROS is produced in duckweed (**Figure 8A**). **Figure 8** showed that ROS production level significantly improved in overexpressed lines compared with WT lines, with a dramatic increase in guard cells of mature fronds. Furthermore, to determine the effect of sucrose on WT duckweed stomata, we measured ROS immediately after treating WT lines with 400 mM sucrose under light for 15 hours (**Figure S5**). **Figure S5** showed that a high concentration of sucrose decreases the stomatal aperture, a process of ROS production. Although LpSUT2 protein was expressed in all tissues, the ROS content of guard cells was significantly increased in overexpressed lines (**Figure 8**), which meant that sucrose stress was formed only in guard cells, but not in other cells. Further, we determined the starch content of duckweed with HPLC, indicating that the starch content of overexpressed lines was significantly higher than that of WT lines (P < 0.001) (**Figure S6**). The starch of duckweed is mainly synthesized in chloroplasts (Guo et al., 2020; Li et al., 2021). However, guard cells possess fewer and smaller chloroplasts (Flütsch et al., 2020) and their starch synthesis ability is weak. Therefore, in overexpression lines, sucrose is synthesized into starch in other cells and formed higher sucrose stress in guard cells, generating the signal molecule ROS and stimulating LpSUT2 local endocytosis of guard cells. Subsequently, the osmotic pressure in guard cells decreased, and the loss of water in guard cells eventually leads to the decrease of stomatal conductance. These results were in accordance with previous studies that sugar elicits stomatal closure, and this process relies on ROS accumulation (Kelly et al., 2013; Lugassi et al., 2015; Li Y. et al., 2018; Martinière et al., 2019). Therefore, our results indicated that ROS might induce the local endocytosis of LpSUT2:eGFP, resulting in eliminating LpSUT2 protein from the PM during the development of young fronds into mature fronds. Also, our result is consistent with previous studies that ROS activate constitutive endocytosis (Zelazny et al., 2007; Hachez et al., 2013; Meng et al., 2017; Martinière et al., 2019; Cui et al., 2021).

## Discussion

**Figure 9:**
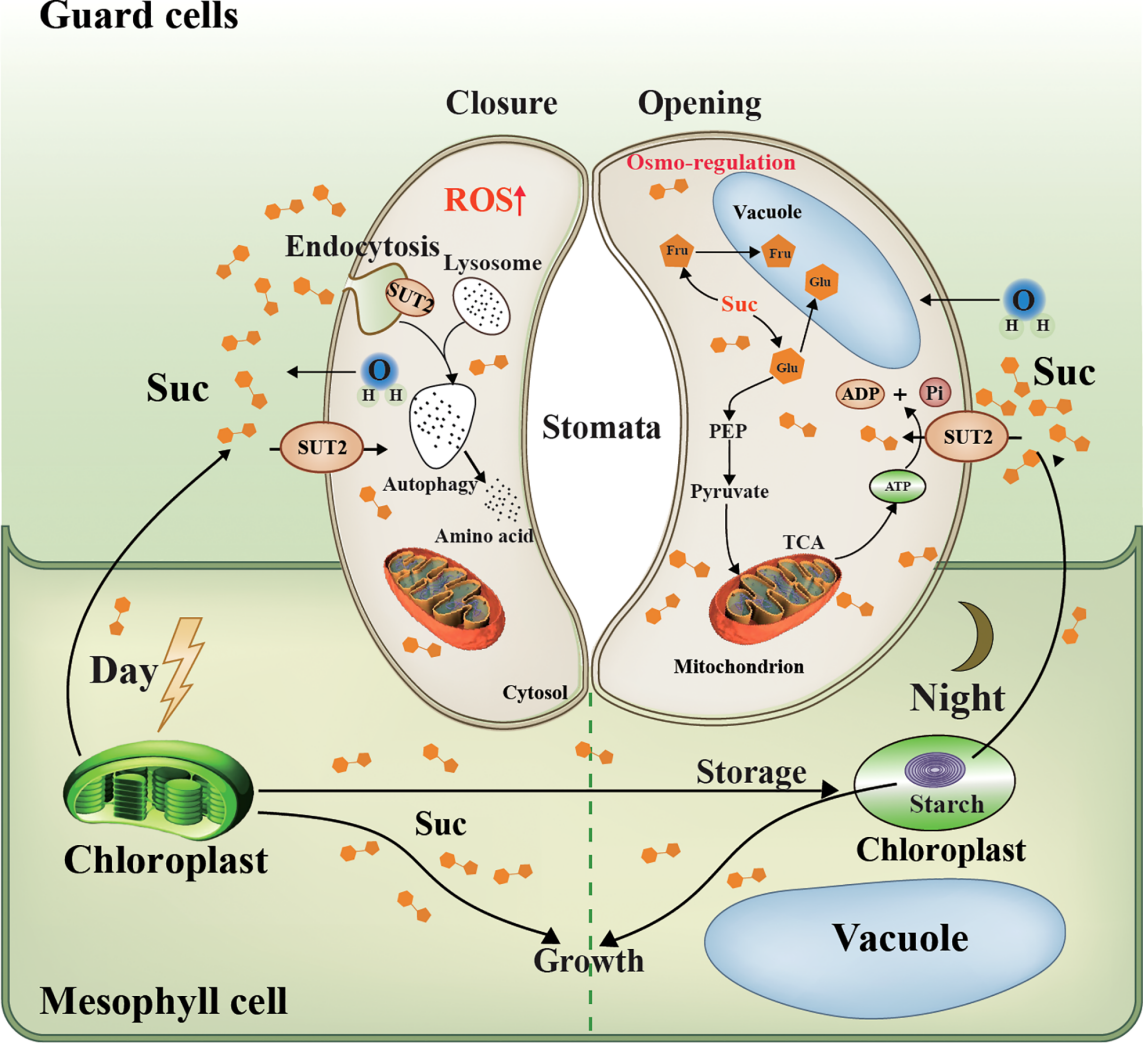
Schematic representation of LpSUT2 local endocytosis in guard cells. Photosynthesis in mesophyll cells is the main source of sucrose for the whole plant (Flütsch et al., 2020). In the daytime, fixed CO_2_, in the form of soluble sugar, is either stored in the chloroplast as transient starch or exported as sucrose to heterotrophic tissues such as root and guard cells (MacNeill et al., 2017). Our work showed that LpSUT2:eGFP protein was polarly distributed in duckweed guard cells: it is only located on the endomembrane of guard cells in the mature fronds of duckweed, while symmetrical distributed on that of young fronds. This might result from the up-regulation of LpSUT2 expression that led to hyperosmotic stress in guard cells, and then triggered the production of the signal molecule ROS, subsequently activated local endocytosis of LpSUT2 in guard cells. The change of stomatal behavior further suggests that the local endocytosis LpSUT2 occurred. Sucrose in guard cells can be broken down into hexose and stored in vacuoles (glucose and fructose), or can be produced into pyruvate through glycolysis, which enters the mitochondria to produce ATP through the TCA cycle. The opening of the stomata consumes energy, the ATP produced from the TCA cycle mentioned above. LpSUT2, sucrose transporter of *L. punctata*; Suc, sucrose; CO_2_, carbon dioxide; eGFP, Enhanced green fluorescent protein; Glu, Glucose; Fru, fructose; Mal, malate; ATP, Adenosine triphosphate; ADP, adenosine diphosphate; TCA, tricarboxylic acid cycle; PEP, phosphoenolpyruvate.

Endocytosis, one of the basic cellular processes, determines the fate of many PM proteins and affects a large number of signal pathways (Mao et al., 2021), the cells’ glucose uptaking (Seaman, 2012), and the maintenance of intracellular metal ions stability (Steinberg et al., 2013; Curnock and Cullen, 2020). In the past few decades, endocytosis of PM proteins has always been a research hotspot (Johnson et al., 2021), such as StSUT1 accumulates in lipid raft-like microdomains under oxidizing conditions (Krugel et al., 2008) and StSUT1 endocytosis and recycling at the plasma membrane (Liesche et al., 2008). In addition, sucrose transporters from peach trees are shown to undergo substrate-induced endocytosis (Zanon et al., 2015). However, the role of local endocytosis of PM protein in guard cells has not yet been researched. In this study, we obtained transgenic duckweed lines with *LpSUT2* gene overexpression, and there was no difference between overexpressed duckweed lines and WT lines, including morphology (frond and root), size, and growth. We constructed a model diagram regarding local endocytosis of guard cells with PM protein LpSUT2 (**Figure 9**). Part of sucrose in mesophyll cells is transported into guard cells to provide osmotic pressure and therefore maintain the stomatal opening in WT lines. The up-regulation of *LpSUT2* expression may stimulate a higher sucrose level, triggering hyperosmotic stresses as it flows into guard cells, then inducing ROS production, which finally activates the local endocytosis LpSUT2 in guard cells. Meanwhile, the stomatal conductance in overexpressed lines decreased during its development. The reduction of stomatal conductance further suggests that the local endocytosis of LpSUT2 occurred in guard cells (**Figure 9**). Our work uncovered the polar localization of LpSUT2 at duckweed guard cells PM.

ROS is one of the second messengers under hyperosmotic stress and accumulates quickly under such conditions (Kilani et al., 2015; Martinière et al., 2019). Various abiotic stresses such as sugar osmotic stress and salt stress (Kilani et al., 2015; Lugassi et al., 2015) induce plants’ accumulation of ROS, including superoxide anion, H_2_O_2_, hydroxyl radical, and singlet oxygen (Zhang et al., 2021), which subsequently activate PM proteins endocytosis (Martinière et al., 2019). Similarly, our results indicated that sucrose content in guard cells might increase and form sucrose stress due to the up-regulation of the *LpSUT2* gene during the initial stage of guard cell development (**Figure 6**). We found that the content of ROS in guard cells of overexpressed lines was higher than that of WT lines (**Figure 8**), which then activated the local endocytosis of LpSUT2 protein in guard cells (**Figure 7A**). Previous research indicated that H_2_O_2_ decreased the density of AtPIP2;1:GFP protein *via* endocytosis on the PM (Wudick et al., 2015), similar to the result of our study. Furthermore, sucrose, an osmotic substance of guard cells, regulates stomatal movement (Granot and Kelly, 2019). The stomatal changes observed in our work further proved the local endocytosis of LpSUT2 in guard cells (**Figure 7**). Hence, we suggested that higher sucrose stress triggered ROS accumulation within guard cells, and then stimulated the local endocytosis of LpSUT2 protein.

The cell wall may affect the dynamic changes of the subcellular localization of LpSUT2 protein. Guard cell walls in plants commonly develop asymmetrically (Carter et al., 2017; Zhang and Dong, 2018). The cell wall tends to be thin and uniform in the young guard cells, with only the primary wall and no secondary wall. Yet the inner wall of mature guard cells stiffens due to the forming of a secondary wall, which reduces the space between the endomembrane and the cell wall (Palevitz, 1981; Carter et al., 2017), and then anchors PM proteins such as aquaporins PIP and auxin efflux transporter PIN (Feraru et al., 2011; Hosy et al., 2015; Łangowski et al., 2016). Interestingly, our results suggested that LpSUT2:eGFP gradually formed polar distribution during the development of guard cells (**Figure 7A**). LpSUT2:eGFP was evenly distributed on the PM at the early stage of development, but only on the endomembrane at the mature stage (**Figure 7A**). Thus, we suspected that the long extracellular domain of LpSUT2 (**Figure 1A**) was anchored on the thickened inner wall, inhibiting the endocytosis of LpSUT2 in the inner membrane region. Other research showed that GFP had a high movement rate in fresh protoplasts, which later decreased by more than 20 times after cell wall regeneration (Martiniere et al., 2012). This result also suggested anchoring the extracellular domain of LpSUT2 on the thickened inner wall. In addition, the cell wall is considered an essential factor in maintaining the polar distribution of PM proteins (Feraru et al., 2011; Łangowski et al., 2016). For example, when the cell wall is disrupted, the lateral diffusion of polar protein (PIN and PIP protein) increases and PIN is no longer polarly distributed (Feraru et al., 2011; Łangowski et al., 2016). Therefore, the extracellular domain of LpSUT2 protein could be anchored on the mature guard cell inner wall to avoid endocytosis.

Polarized growth is a fundamental biological process during plant development and requires precise spatial and temporal control of PM protein localization. Plant stomata development exhibits a polar growth pattern (Carter et al., 2017; Wang et al., 2022), similar to the plant root hair and pollen tube (Li H. et al., 2018; Zhu et al., 2020; Kuběnová et al., 2022). In our results, local endocytosis rather than constitutive endocytosis occurred on the PM protein LpSUT2 in guard cells that showcases polar growth (**Figure 7A**). This local endocytosis can keep the wholeness of the guard cells’ inner wall and help maintain the morphology, function, and polarity of the guard cell. Therefore, we found a potential polar protein that regulates the changes of stomatal behavior by local endocytosis in duckweed guard cells.

## Conclusions

In conclusion, we found that duckweed LpSUT2 protein, a transmembrane protein, is highly expressed on guard cells PM and uncovered the role of ROS-dependent LpSUT2 local endocytosis in the development of overexpressing duckweed. Changes in duckweed stomatal behavior proved that ROS-dependent local endocytosis occurred on LpSUT2 in guard cells. The genetic manipulation of the *LpSUT2* gene reduces stomatal conductance and may provide a powerful strategy to improve plant water use efficiency, battle against pathogen invasion, and prevent insect feed or oviposition (Lin et al., 2022), as well as offer a theoretical basis for applications in crops.

## Data availability statement

The original contributions presented in the study are included in the article/**Supplementary Material**. Further inquiries can be directed to the corresponding author.

## Author contributions

PL: Investigation, Methodology, Software, Visualization, Writing-original draft, Writing-review & editing; YF, YJ, ZY: Funding acquisition, Methodology, Project administration, Writing-review & editing; KH: Methodology, Project administration, Writing-review & editing; XT: Investigation, Methodology, Formal analysis, Writing-review & editing; ZH: Formal analysis, Writing-review & editing; CW: Formal analysis, Writing-review & editing; RC: Writing-review & editing; HZ: Conceptualization, Funding acquisition, Project administration, Resources, Supervision, Writing-review & editing. All authors contributed to the article and approved the submitted version.

## Funding

This study was supported by Innovation Academy for Seed Design, CAS; National Aquatic Biological Resource Center (NABRC); CAS “Light of West China” Program (2020XBZG_XBQNXZ_A_001); CAS “Light of West China” Program (2018XBZG_XBQNXZ_B_007) and Biological Resources Programme, Chinese Academy of Sciences (KFJ-BRP-008).

## Acknowledgments

We thank Leyi Zhao for providing the Writing-review and editing.

## Conflict of interest

The authors declare that the research was conducted in the absence of any commercial or financial relationships that could be construed as a potential conflict of interest.

## Publisher’s note

All claims expressed in this article are solely those of the authors and do not necessarily represent those of their affiliated organizations, or those of the publisher, the editors and the reviewers. Any product that may be evaluated in this article, or claim that may be made by its manufacturer, is not guaranteed or endorsed by the publisher.
