# Supplementary material for: Local endocytosis of sucrose transporter 2 in duckweed reveals the role of sucrose transporter 2 in guard cells
Source: Front Plant Sci. 2022 Oct 24;13:996618. doi: 10.3389/fpls.2022.996618 (PMC9638040; doi:10.3389/fpls.2022.996618)
Supplement: Supplementary file 1 [file DataSheet_1.pdf]

Supplementary Material

Supplementary figures

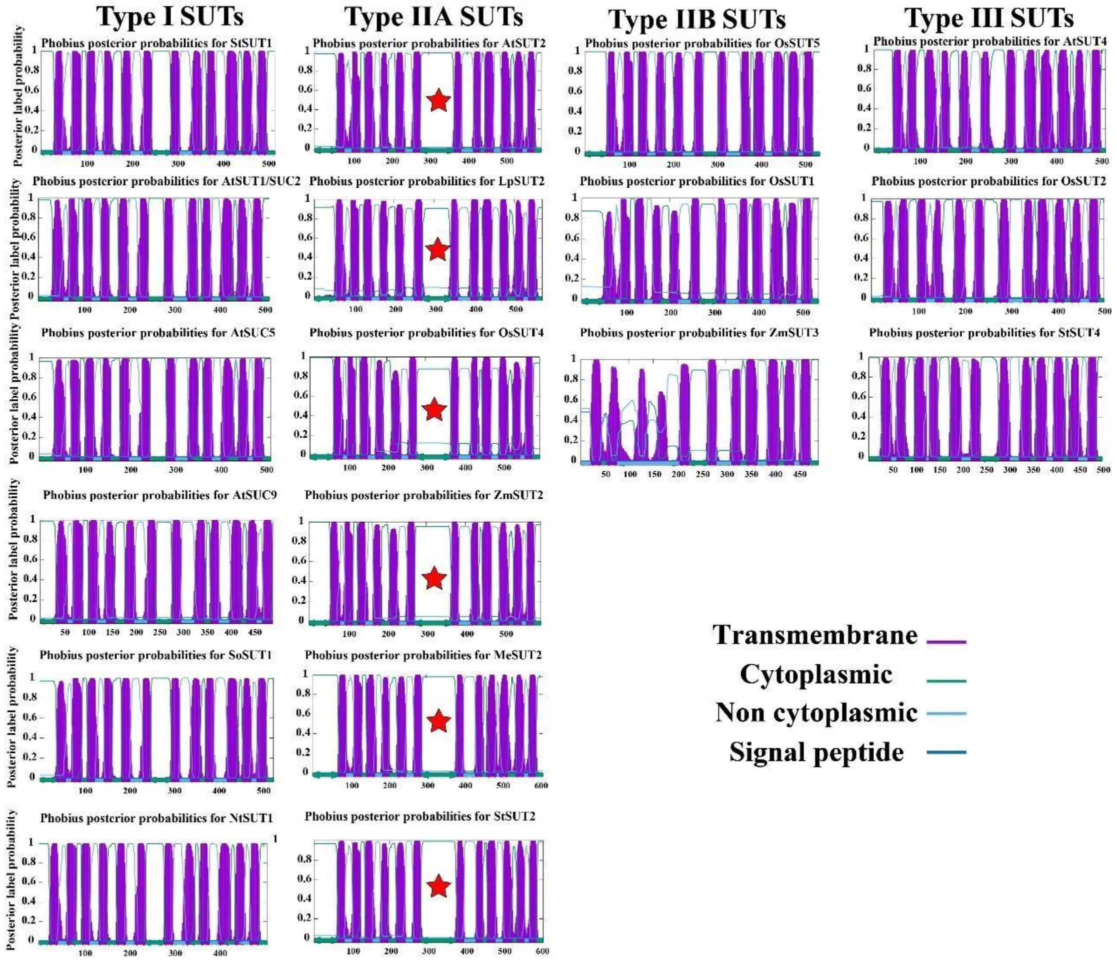

Supplementary Figure 1. The transmembrane domains of SUTs. The long central cytoplasmic loops of type IIA SUTs members are displayed (Marked by red stars). The figure was produced using the Phobius server (https://phobius.sbc.su.se/) and Gnuplot software.

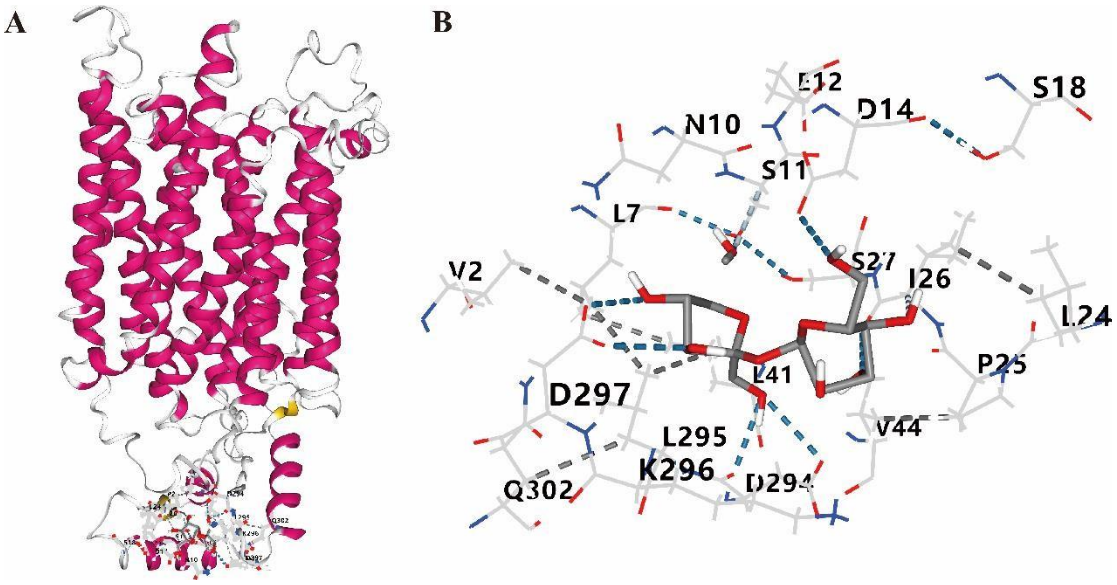

Supplementary Figure 2. Molecular docking of LpSUT2 protein and sucrose. We predicted the binding site of LpSUT2 protein and sucrose by CB-Dock database. **(A).** The molecular docking graph of LpSUT2 protein and sucrose. **(B).** The key amino acids in the N-terminal sequence of LpSUT2 protein.

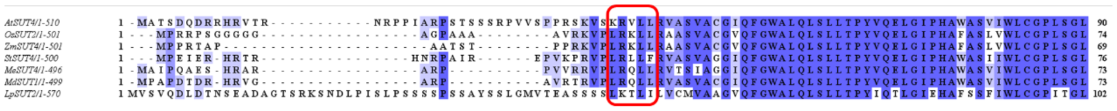

Supplementary Figure 3. Putative vacuolar targeting motif (LXXLL) in Type III SUTs and LpSUT2 displayed by amino acid sequence alignment using Jalview software. Amino acid conserved sites that conform to the motif LXXLL are marked in the red box. Abbreviations: LpSUT2, Sucrose transporter 2 of *L. punctata*.

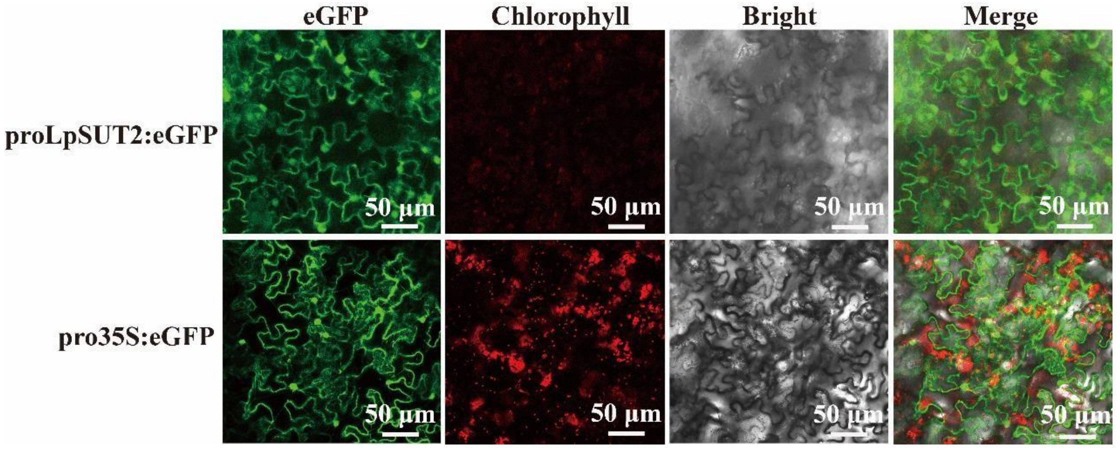

Supplementary Figure 4. LpSUT2 promoter activity analyzed through transient expression in *Nicotiana tabacum* leaves. The binary vector pCambia2301:pro35S:eGFP (Positive control) and pCambia2301:proLpSUT2:eGFP were transiently expressed in *Nicotiana tabacum* leaves and then observed under a laser scanning confocal microscope (excitation filter, 488 nm; emission filter bandpass, 505-530 nm). Fluorescence images present that the strength of LpSUT2 promoter is almost equal to that of 35S promoter. The eGFP protein is indicated in green, and chlorophyll autofluorescence is indicated in red. The eGFP, chlorophyll

auto-fluorescence, bright field, and merged images were presented. Scale bars: 50 μm. Abbreviations: LpSUT2, Sucrose transporter 2 of *L. punctata*; eGFP, Enhanced green fluorescent protein.

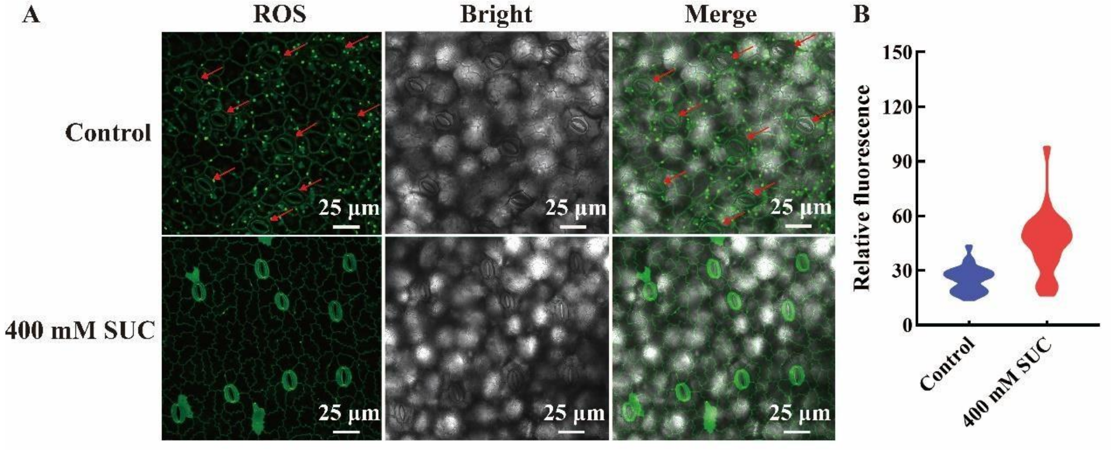

Supplementary Figure 5. Effects of sucrose (400 mM) on stomatal apertures in *Lemna minor*. ROS was measured immediately after being treated with 400 mM sucrose for 15 h under light treatment. These results showed that high sucrose concentration induced the decrease of stomatal aperture, which depended on the accumulation of ROS. **(A)**. At least 40 guard cells were quantified under a laser scanning confocal microscope. The fluorescence intensity of ROS of the treatment group was higher than that of the control group **(B)**. The red arrow points to the guard cell. Scale bars: 25 μm. Abbreviations: ROS, Reactive oxygen species.

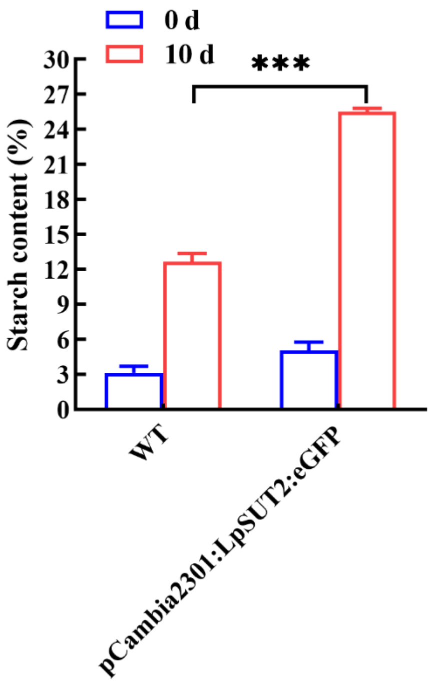

Supplementary Figure 6. The determination of starch content in duckweed. We detected the starch content of WT lines and overexpressed lines after culturing for 10 d under light conditions. The starch content was determined using an evaporative light-scattering detector (All-Tech ELSD 2000, All-tech, Crop, USA) of HPLC

(highperformance liquid chromatography) system (Thermo 2795, Thermo Corp, USA). The results showed that the starch content of overexpressed lines was 2.01 times of that of WT lines after culturing for 10 d under light conditions. The bars indicate the mean value ± SD of three independent experiments (***$P < 0.001$). Abbreviations: WT, Wild type.

Supplementary Table 1. Proteins used in the phylogenetic tree of SUTs.

Protein name	Plant species	Accession number
AtSUC1	*Arabidopsis thaliana*	AT1G71880
AtSUT1/SUC2	*Arabidopsis thaliana*	AT1G22710
AtSUT2	*Arabidopsis thaliana*	AT2G02860
AtSUT4	*Arabidopsis thaliana*	AT1G09960
AtSUC5	*Arabidopsis thaliana*	AT1G71890
AtSUC6	*Arabidopsis thaliana*	AT5G43610
AtSUC7	*Arabidopsis thaliana*	AT1G66570
AtSUC8	*Arabidopsis thaliana*	AT2G14670
AtSUC9	*Arabidopsis thaliana*	AT5G06170
OsSUT1	*Oryza sativa Japonica Group*	AAF90181.1
OsSUT2	*Oryza sativa Japonica Group*	AAN15219.1
OsSUT3	*Oryza sativa Japonica Group*	BAB68368.1
OsSUT4	*Oryza sativa Japonica Group*	BAC67164.1
OsSUT5	*Oryza sativa Japonica Group*	BAC67164.1
BoSUT1	*Zea mays*	100273131
ZmSUT5	*Zea mays*	PWZ27816.1
ZmSUT3	*Zea mays*	PWZ57257.1
ZmSUT1	*Zea mays*	BAA83501.1
ZmSUT2	*Zea mays*	AAS91375.1
ZmSUT4	*Zea mays*	AAT51689.1
ZmSUC3	*Zea mays*	AQL09360.1
StSUT1	*Solanum tuberosum*	CAA48915.1
StSUT2	*Solanum tuberosum*	AAP43631.1
StSUT4	*Solanum tuberosum*	AAG25923.2
TaSUT1A	*Triticum aestivum*	AAM13408.1
TaSUT1B	*Triticum aestivum*	AAM13409.1
TaSUT1B	*Triticum aestivum*	AAM13410.1
ShSUT1	*Saccharum hybrid cultivar*	AAV41028.1
NtSUT1	*Nicotiana tabacum*	CAA57727.1

NtSUT3	*Nicotiana tabacum*	AAD34610.1
MeSUT2	*Manihot esculenta*	ABA08445.1
MeSUT4	*Manihot esculenta*	ABA08443.1
MdSUT1	*Malus domestica*	AAR17700.1
HbSUT3	*Hevea brasiliensis*	ABK60190.2
LeSUT1	*Solanum lycopersicum*	CAA57726.1
SoSUT1	*Spinacia oleracea*	CAA47604.1
SpSUT4	*Spirodela polyrrhiza*	Si2G0106800
SpSUT2	*Spirodela polyrrhiza*	Spi11G0051900
LmSUT4	*Lemna minor*	Lmi017542
LmSUT2	*Lemna minor*	Lmi005811
LpSUT2	*Landoltia punctata*	Lp10005267
